# Effect of Side Chain Functional Groups on the DPPH Radical Scavenging Activity of Bisabolane-Type Phenols

**DOI:** 10.3390/antiox8030065

**Published:** 2019-03-16

**Authors:** Kazuya Ichikawa, Ryosuke Sasada, Kosuke Chiba, Hiroaki Gotoh

**Affiliations:** Department of Chemistry and Life Science, YOKOHAMA National University, 79-5 Tokiwadai, Hodogaya-ku, Yokohama 240-8501, Japan; ichikawa-kazuya-ym@ynu.jp (K.I.); sasada-ryosuke-cy@ynu.jp (R.S.); chiba-kosuke-dh@ynu.jp (K.C.)

**Keywords:** curcuphenol, electron proton transfer, phenols, sesquiterpenes, xanthorrhizol

## Abstract

Methods for improving the antioxidant activity of phenolic compounds have been widely investigated; however, most studies have focused on the structure–activity correlations of substituents on the aromatic rings of catechols or flavonoids. We investigated the influence of side chain functional groups on the 2,2-diphenyl-1-picrylhydrazyl (DPPH) radical scavenging activity of xanthorrhizol and curcuphenol analogues. These compounds were synthesised by the side chain functional group conversion of curcumene, followed by direct oxidation of the aromatic ring. We determined the DPPH radical scavenging activity from the half-maximal effective concentration (EC_50_) obtained from a DPPH assay in methanol. The positional relationships of the side chain with the aromatic ring and phenolic OH group were determined using density functional theory calculations, and the stability of different conformations was compared. Electron transfer-proton transfer was determined to be the dominant mechanism in the DPPH reaction with xanthorrhizol analogues, based on the correlation between the EC_50_ and ionisation potential. The radical cation was greatly stabilised in the structure where the side chain functional group was close to the aromatic ring. Stabilisation also depended on the phenolic OH group position. In future antioxidant design, aromatic ring substituent conversion and the use of functional groups far from the OH group or ring should be explored.

## 1. Introduction

Some of the oxygen taken into a living body by respiration is converted into reactive oxygen species (ROS), which are considered to contribute to cancer and aging due to the damage they cause to nucleic acids, lipids, and proteins [1,2]. Phenolic compounds are well known for reducing and detoxifying ROS [3,4] through a hydrogen atom transfer mechanism (HAT, Equation (1)) [5], a single electron transfer mechanism (electron transfer-proton transfer, ET-PT, Equation (2)) [6], or a sequential proton loss electron transfer mechanism (SPLET, Equation (3)) [7,8]. It has been shown that these radical scavenging activities of phenolic antioxidants are related to the phenolic O–H bond dissociation enthalpy (BDE), ionization potential (IP), proton dissociation enthalpy (PDE), proton affinity (PA), and electron transfer enthalpy (ETE). It is therefore indispensable to study the structure–activity correlation and reduction mechanism of DPPH radicals of phenolic compounds in order to improve their DPPH radical scavenging activity.

(1) ArOH+DPPH•→ArO•+DPPH−H

(2a) $$\mathrm{ArOH}+\mathrm{DPPH}•\to \mathrm{ArOH}{•}^{+}+{\mathrm{DPPH}}^{-}$$

(2b) $$\mathrm{ArOH}{•}^{+}+{\mathrm{DPPH}}^{-}\to \mathrm{ArO}•+\mathrm{DPPH}-H$$

(3a) $$\mathrm{ArOH}\to {\mathrm{ArO}}^{-}+H^{+}$$

(3b) $${\mathrm{ArO}}^{-}+\mathrm{DPPH}•\to \mathrm{ArO}•+{\mathrm{DPPH}}^{-}$$

Electronic factors for improvements to the antioxidant activity of catechols and benzylphenols have been studied with DPPH [9] assays and computational chemistry using analogues with new substituents on the aromatic ring [10]. A comprehensive structure–activity correlation by substituent conversion of phenolic and aniline compounds has recently been reported [11]. Intramolecular interactions due to substituents adjacent to phenolic OH groups as reaction sites have also been found to contribute to the stabilisation of intermediates, as exemplified by studies with catechols [12,13]. Moreover, it has been established that antioxidant behaviour cannot be fully rationalized unless interactions with the surrounding medium are carefully considered. The role of the side chain in antioxidant activity has also been studied previously, considering its importance in the polarity, mobility, and localisation of the antioxidant [14].

**Figure 1:**
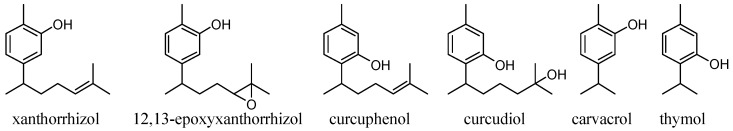
Bisabolane-type sesquiterpenoids and similar phenols from natural products.

Phenolic natural products with a bisabolane-type sesquiterpene skeleton [15,16,17] (Figure 1) have been isolated from marine sponge organisms and fern plants, and have various antioxidant activities, depending on the substitution position of the phenolic OH group or the type of functional group on the side chain. In a study of the suppression of the production of lipid peroxide using rat brain homogenate as a reaction field, curcuphenol showed moderate activity, of the 11 tested phenolic metabolites extracted from marine invertebrates. However, curcudiol, a compound with an OH group on the side chain, had the highest antioxidant activity [18]. Moreover, in the evaluation of the antioxidant activity in cells using a cell permeable fluorescent probe (DCFH–DA), curcudiol showed activity while curcuphenol did not [19]. It has been confirmed in other compounds such as hydroquinones that antioxidant activity greatly changes when an OH group is present on the side chain [20]. Xanthorrhizol (**1**) has drawn attention from drug discovery researchers [21] because it has numerous beneficial physiological activities such as neuroprotective ability [22], lipid peroxidation inhibitory ability (antioxidant ability [22,23]), and antibacterial ability [24,25]. Acetylation of the phenolic OH in xanthorrhizol (**1**) reduces its physiological activity, strongly suggesting that the phenolic OH group is involved in the physiological activity mechanism [22,23,24]. The influence of functional groups on the side chain, such an OH group, has not been studied for xanthorrhizol (**1**) analogues.

Evaluation of the antioxidant activity of compounds such as xanthorrhizol (**1**) has been carried out using DPPH assays and lipid peroxide suppression reactions [21,22,23,24]. Analytical evaluation of compounds with different side chain functional groups in the same skeleton showed a difference in antioxidant activity, but the influence of the side chain on a compound’s antioxidant activity has rarely been discussed. If the influence of the side chain functional group on the compound’s antioxidant activity can be clarified, that information would be very useful in the molecular design of new antioxidants.

In this study, curcumene, which can be synthesised in two steps from inexpensive reagents, was used as a starting substrate. After converting the side chain, aromatic ring oxidation by phthaloyl peroxide was performed to synthesise multiple analogues, especially xanthorrhizol (**1**) analogues, which are relatively more difficult to synthesise than curcuphenol analogues. In order to investigate the influence of the side chain on the DPPH radical scavenging activity, the synthesised analogues, as well as commercially available carvacrol and thymol, which do not have side chains, were evaluated by DPPH assays. We also clarified the influence that the side chain has on the reduction mechanism based on the correlation between thermodynamic parameters obtained by density functional theory (DFT) calculations and DPPH assay values. There is no prior research that has used theoretical calculations to evaluate the effect of side chain functional groups on the DPPH radical scavenging activity of curcumene derivatives; however, discussions based on experimental results and calculations can be expected in the future development of such research.

## 2. Materials and Methods

### 2.1. Synthesis

Compounds with bisabolane-type sesquiterpene skeletons were synthesised by side chain conversion from curcumene, which can be synthesised using the reported methods, followed by direct oxidation of the aromatic rings using phthaloyl peroxide [26]. Side chain conversion was used not only to synthesise olefins but also to append tertiary OH, epoxy, and ketone groups.

### 2.2. Measurement of EC_50_ with DPPH Assay

DPPH radical scavenging activity was measured as described previously [27], with a slight modification. Each phenol was dissolved in methanol to make 5 mL of each solution, ranging in concentration from 0.10 to 5.60 mM. A solution of DPPH (4.4 mg) in methanol (4.41 mL) was also prepared. The DPPH solution (0.10 mL) was added to each phenol solution, and after 30 min, the absorbance of the mixtures and the blank was measured at 517 nm using a V-550 (cell length: 1 cm) UV–Vis spectrophotometer (JASCO Corporation, Tokyo, Japan). From the change in absorbance (Abs) at 517 nm, the reduction rate of each compound was calculated using the following formula:

$$\mathrm{Reduction}\;\mathrm{rate}\;\left(\%\right)=\frac{{\mathrm{Abs}}_{blank}-{\mathrm{Abs}}_{sample}}{{\mathrm{Abs}}_{blank}}\times 100.$$

After plotting the reduction rate vs. concentration (SI), the half-maximal (50%) effective concentration (EC_50_) of each compound was calculated using a straight line taken from two points crossing the 50% reduction rate. As a model substrate, commercially available reagents without any side chains, thymol and carvacrol, were also evaluated using the same method.

### 2.3. Theoretical Calculations

A single-point calculation in methanol was carried out for the geometry at the (U)B3LYP/6-311+G** level. IEF-PCM was used to take the solvent effect into account. We used the most stable structure of each compound, charge and multiplicity, and distance feature to calculate the thermodynamic parameters.

To elucidate the influence of the side chain’s functional groups on the DPPH radical scavenging activity of each synthesised compound, a conformation search of each synthesised compound in the neutral, radical, radical cation, and anion form was performed by molecular force field calculations using a Molecular Mechanics program 3 (MM3) [28,29]. Structures in which the functional groups on the side chain are close to the phenolic OH group or the aromatic ring, and one in which the functional groups on the side chain are far from them are defined as the initial structures. Energy was calculated using DFT calculations for 3–4 conformations in each case where the side chains were close and far. These structures were used to perform DFT calculation with the Gaussian 16 program [30]. Calculations were carried out at the (U)B3LYP/6-31G* level for gas-phase structural optimisation, and at the B3LYP/6-311+G** level with integral equation formalism-polarisable continuum models (IEF-PCM) [31] for the enthalpy of each compound in methanol. However, it should be noted that with the method using IEF-PCM, it is not possible to consider cases where hydrogen bonding occurs with solvent molecules. Using these calculation results, stability was compared based on the enthalpy when the side chains were close and far. Based on the enthalpy of each stable structure, five thermodynamic parameters, i.e., bond dissociation enthalpy (BDE), ionisation potential (IP), proton dissociation enthalpy (PDE), proton affinity (PA), and electron transfer enthalpy (ETE), were calculated using the following formulas. The BDE values were used to estimate the reactivity of an ArOH in HAT. The IP and PDE values from the ArOH•^+^ radical cation were calculated to describe the ET-PT mechanism. The PA values of the phenoxide anion, ArO^−^, were used to characterise the reaction enthalpy of the first step, and ETE for the reaction enthalpy of the following step in the SPLET mechanism. The values H(e^−^) = −19.85 kcal/mol and H(H^+^) = −246.6 kcal/mol were used as the enthalpies of protons and electrons in methanol, respectively [32,33,34]. The Winmostar program [35] was used to visualise each structure.

BDE=H(ArO•)+H(H•)−H(ArOH)

$$\mathrm{IP}=H\left(\mathrm{ArOH}{•}^{+}\right)+H\left(e^{-}\right)-H\left(\mathrm{ArOH}\right)$$

$$\mathrm{PDE}=H\left(\mathrm{ArO}•\right)+H\left(H^{+}\right)-H\left(\mathrm{ArOH}{•}^{+}\right)$$

$$\mathrm{PA}=H\left({\mathrm{ArO}}^{-}\right)+H\left(H^{+}\right)-H\left(\mathrm{ArOH}\right)$$

$$\mathrm{ETE}=H\left(\mathrm{ArO}•\right)+H\left(e^{-}\right)-H\left({\mathrm{ArO}}^{-}\right)$$

## 3. Results

### 3.1. Synthesis

**Scheme 1:**
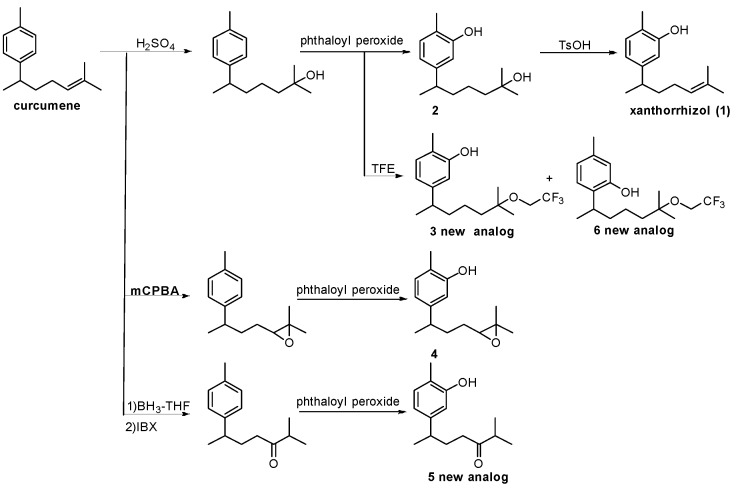
Synthesis of bisabolane-type phenolic sesquiterpenes. TFE: 2,2,2-trifluoroethanol; mCPBA: *m*-chloroperoxybenzoic acid; IBX: 2-iodoxybenzoic acid; BH_3_-THF: borane-tetrahydrofuran complex.

Six phenolic compounds with a bisabolane-type sesquiterpene skeleton, including three novel analogues (compounds **3**, **5**, and **6**), were synthesised (Scheme 1). Xanthorrhizol (**1**) derivatives were predominantly synthesised over curcuphenol derivatives during the direct oxidation step. The regioselectivity of the oxidation was a result of a preferential reaction on the side with less steric hindrance, which concurs with a previous study [26]. Direct conversion from curcumene to xanthorrhizol was difficult as the olefin was oxidised earlier than the aromatic ring. For this reason, after the olefin was converted to a tertiary OH, the aromatic ring was oxidised and the tertiary OH was converted back to an olefin. The commercially available compounds thymol and carvacrol, containing no side chains, were used as model substrates.

### 3.2. Theoretical Calculations

The enthalpy differences between the compounds, depending on whether the side chain functional group is in a close conformation or far conformation, are shown in Table 1. Compounds **1**–**5** tend to be more stable in a far conformation, with the exception of some of the radical cations, whereas the radical cation and anion of compound **6** are more stable in a close conformation. The five thermodynamic parameters determined from the DFT calculations are shown in Table 2, together with the EC_50_. Compound **6**, containing the highest radical scavenging ability, exhibits low values for the BDE and ETE, and xanthorrhizol (**1**) has shown a low value for the IP.

### 3.3. Measurement of EC_50_ with DPPH Assay

The results of the DPPH assay are shown in Table 2. Thymol had a higher radical scavenging ability than carvacrol, which is consistent with the results reported previously [36]. Compound **6** showed the highest radical scavenging ability. Xanthorrhizol (**1**) had the highest radical scavenging ability among the xanthorrhizol derivatives (compounds **1**–**5**). Comparing compounds **3** and **6** showed that they had the same side chain functional groups but a different position of the phenolic OH group; however, the curcuphenol derivative **6** showed higher radical scavenging ability than compound **3**.

## 4. Discussion

### 4.1. Influence of the Side Chain Functional Groups

**Figure 2:**
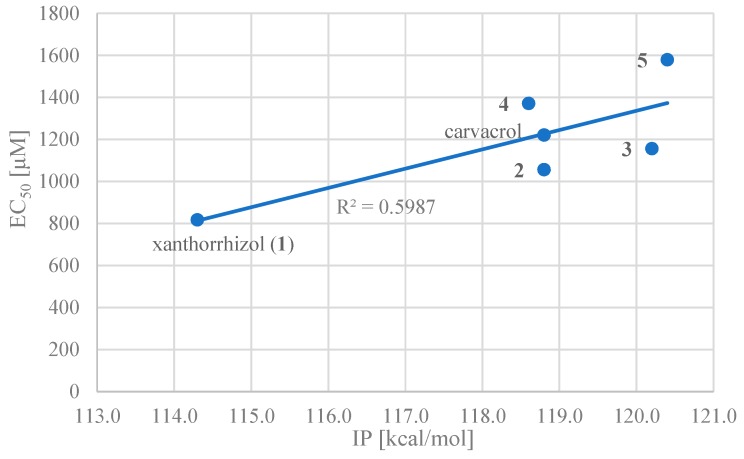
Half-maximal effective concentration (EC_50_) vs. ionisation potential (IP) (compounds **1**–**5** and carvacrol).

Correlations between each thermodynamic parameter and the EC_50_ were obtained for carvacrol and compounds **1**–**5**. The strongest positive correlation (*R*^2^ = 0.60) was that of IP (Figure 2). This suggests that IP, and therefore the ET-PT mechanism, is the largest contributor to the DPPH radical scavenging activity of these compounds. This agrees with a previous study that showed that phenolic compounds react through ET-PT mechanisms in polar solvents such as water and alcohols [37,38]. However, in the future, it should be noted that the substrate for generalisation and more accurate calculations need to be expanded in order to reduce uncertainty and errors.

**Figure 3:**
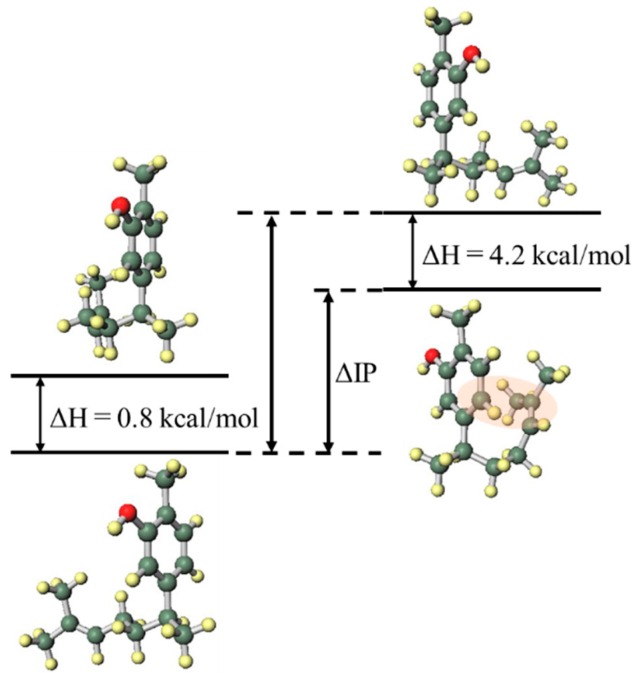
Neutral (**left**) and radical cation (**right**) energy diagrams of xanthorrhizol (**1**).

**Figure 4:**
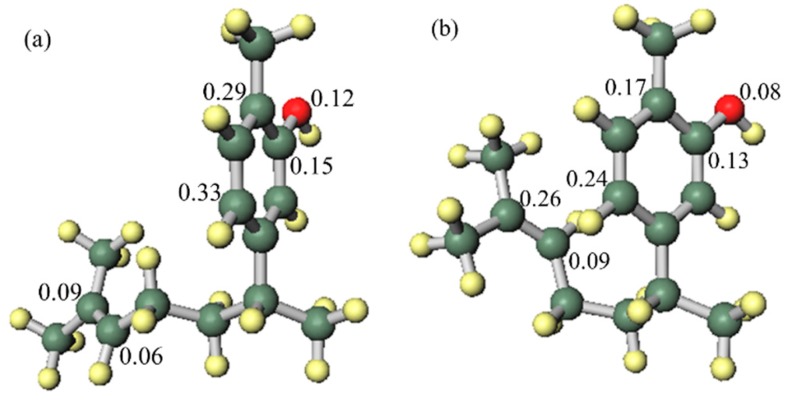
Spin density of the xanthorrhizol (**1**) radical cation: (**a**) far side chain conformation, (**b**) close side chain conformation.

**Figure 5:**
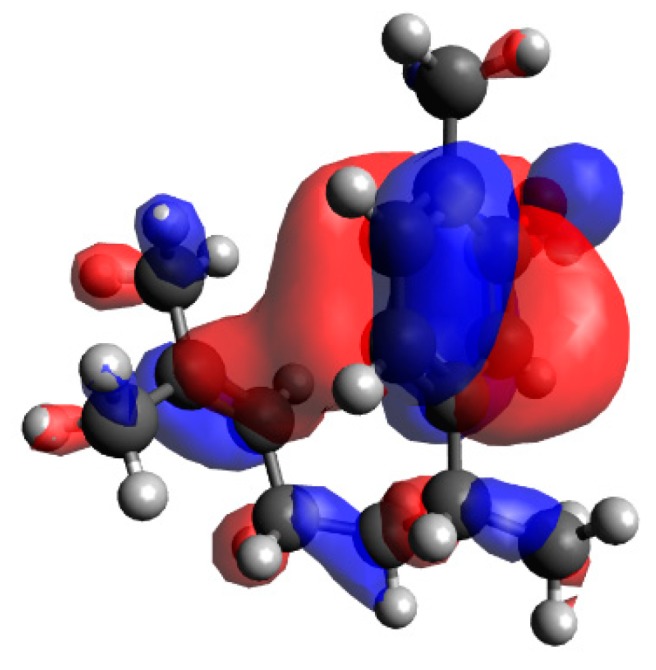
Molecular orbital lobe diagram of the xanthorrhizol (**1**) radical cation (next highest occupied molecular orbital).

In order to investigate the influence of the side chain on the DPPH radical scavenging activity, the thermodynamic stability of the neutral compound, the radical cation and the positional relationship of the side chain were determined. In the case of xanthorrhizol (**1**), which has a high radical scavenging ability and the smallest IP of compounds **1**–**5**, the neutral compound is more thermodynamically stable when the side chain is in the far conformation, but the difference in stability compared to when the side chain is in close conformation is less than 1 kcal/mol. Therefore, the conformation of the side chain does not have a notable influence on the neutral compound. Conversely, the radical cation is more stable when the side chain is in the close conformation, and there is a difference in energy of 4.2 kcal/mol compared with when the side chain is in the far conformation. Therefore, it is conceivable that the functional group on the side chain contributes to the stabilisation of the radical cation (Figure 3). As can be seen in the electron spin density of the radical cation, unpaired electrons are localised in the aromatic ring in the far conformation, whereas unpaired electrons are delocalised into the double-bond moiety on the side chain in the close conformation (Figure 4). In addition, the π orbital of the aromatic ring is stabilised when the side chain is in the close conformation because electrons flow into the aromatic ring from the double-bond moiety of the electron-rich side chain, as can be seen in the orbital lobe diagram (Figure 5). It can therefore be inferred that xanthorrhizol (**1**) exhibited the smallest IP because of stabilisation of the radical cation through the double-bond moiety on the side chain, and as a result, had a higher radical scavenging ability.

### 4.2. Influence of the Phenolic OH Group Position

**Figure 6:**
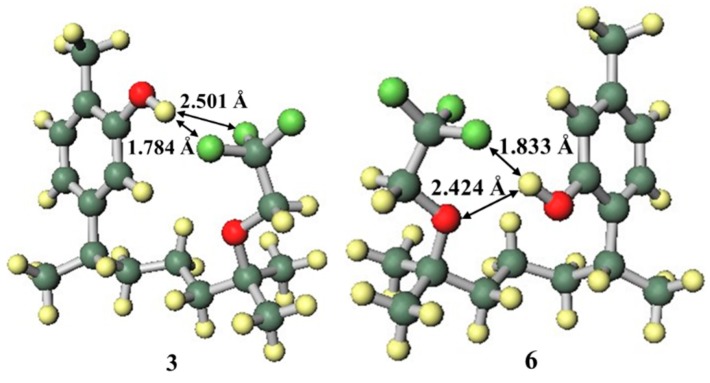
Structures of the radical cations of compounds **3** and **6**.

Carvacrol, thymol, and the new synthetic fluoro analogues **3** and **6** were compared in order to evaluate the influence of the phenolic OH group position. Compounds **3** and **6** had lower EC_50_ values than carvacrol and thymol, respectively. This suggests that the presence of the trifluoroethoxy group on the side chain improves the radical scavenging ability. When the functional group on the side chain was in the close conformation (Figure 6), the radical cation forms of compounds **3** and **6** were stable. However, there is a possibility that the mechanism may completely change due to competition between the groups on the side chains in their interactions, such as hydrogen bonding and intermolecular interaction, with solvent molecules [39,40]. However, intramolecular hydrogen bonding was observed by calculation using the IEF-PCM. Since the fluorine and oxygen atoms of the trifluoroethoxy group stabilised the radical cation by forming hydrogen bonds with the highly cationic hydrogen atom of the phenolic OH group and distortion did not occur, the close conformation was the most stable. This additional stability from the side chain functional group was the reason why compounds **3** and **6** had better EC_50_ values than carvacrol and thymol.

The difference in EC_50_ values between compounds **3** and **6** was larger than that between carvacrol and thymol. Since the side chain functional groups were the same for each pair, it can be inferred that the positional relationship between the phenolic OH group and the side chain was the cause of this difference. As can be seen in Table 1, while compound **3** was stabilised by a close trifluoroethoxy group on the side chain only for the radical cation, compound **6** had greater stabilisation for the radical cation and also had stabilisation by a close conformation side chain for the anion.

**Figure 7:**
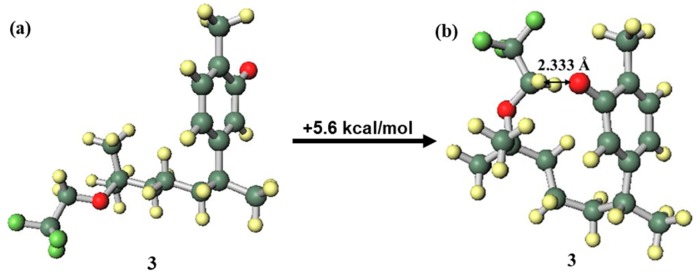
Difference in conformation stability of the compound **3** anion: (**a**) far conformation, (**b**) close conformation.

**Figure 8:**
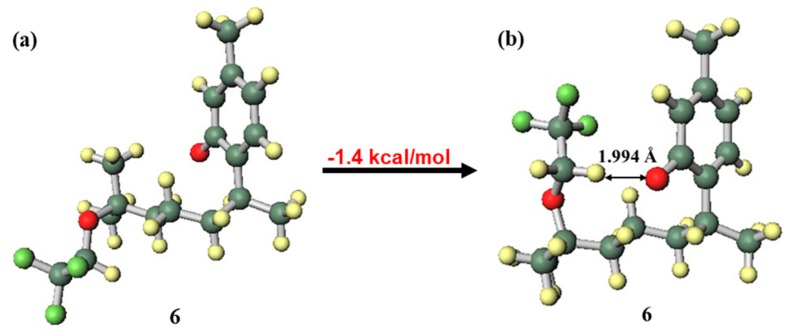
Difference in conformation stability of the compound **6** anion: (**a**) far conformation, (**b**) close conformation.

In the structure of the compound **3** anion (Figure 7a), where the side chain was in the close conformation (Figure 7b), hydrogen bonding between a phenolic oxygen atom with anionic properties and the highly acidic hydrogen atom of a methylene in the trifluoromethoxy group was expected to occur. This hydrogen bond would contribute to stabilisation; however, in compound **3**, this hydrogen bond was formed with distortion generated in the side chain and aromatic ring. This caused destabilisation, and as a result, the far conformation was the more stable structure. In contrast, since the compound **6** anion can form a similar stabilizing hydrogen bond without distortion (Figure 8), the close conformation was the more stable structure. The fact that compound **6** was more likely than compound **3** to be found in an anionic state because of hydrogen bond stabilisation indicates the increased possibility of radical scavenging progressing through a SPLET mechanism [5,39,40], which could be a factor in the good EC_50_ value of compound **6**. To elucidate the mechanism in more detail, it will be necessary to further investigate curcuphenol analogues.

## 5. Conclusions

We synthesised bisabolane-type sesquiterpenoids and novel analogues, and investigated the influence of the side chain functional groups on the DPPH radical scavenging activity of these compounds using a DPPH assay and computer calculations. For compounds **1**–**5**, the IP was the thermodynamic property that made the largest contribution to the radical scavenging ability, and as a result, the ET-PT mechanism was considered to be the dominant reduction mechanism. In addition, the positional relationship between the side chain and the aromatic ring or phenolic OH group was determined to be a cause of decreases in the IP value. A representative example of this is xanthorrhizol (**1**), which had a particularly small EC_50_ value, and was stabilised by the close conformation in the radical cation, and thus the electrons were delocalised due to the functional group on the side chain. Finally, the influence of the phenolic OH group position on the DPPH radical scavenging activity was investigated. It was determined that the magnitude of the stabilisation due to the interaction between the side chain functional group and the phenolic OH group, and the destabilisation due to the distortion of that structure greatly depended on the position of the phenolic OH group. In the case of compound **6**, the stabilisation of the structure by the side chain in the anion led to the improvement of the radical scavenging ability through the SPLET mechanism. Based on these results, not only aromatic ring substituent conversion, but also the possibility of a new approach using functional groups far from the OH group or ring, should be investigated in future antioxidant designs. In the future, we would like to clarify the effects of the side chain by confirming the detailed solvent effect, using more accurate calculations, and measuring the antioxidant activity using methods close to the biological environment, while increasing the substrate range.

## Figures and Tables

**Table 1:** Enthalpies of the close conformation minus the far conformation (kcal/mol).

Compounds	Neutral	Radical	Radical Cation	Anion
xanthorrhizol (**1**)	+0.76	+0.27	−4.2	+3.7
**2**	+3.0	+5.7	−0.33	+2.6
**3**	+2.6	+2.4	−1.1	+5.3
**4**	+1.4	+0.95	+3.4	+2.3
**5**	+0.70	+1.6	+1.1	+2.0
**6**	+2.4	+2.3	−3.9	−1.4

**Table 2:** Thermodynamic parameters (kcal/mol) in methanol and DPPH radical scavenging ability (μM)

Compounds	BDE	IP	PDE	PA	ETE	EC_50_
xanthorrhizol (**1**)	79.6	114.3	12.5	49.9	76.9	817
**2**	79.2	118.8	7.6	49.5	76.9	1056
**3**	80.3	120.2	7.3	50.1	77.3	1156
**4**	79.7	118.6	8.3	50.3	76.6	1371
**5**	80.0	120.4	6.8	50.2	77.0	1579
**6**	78.3	119.4	6.1	50.6	74.9	531
carvacrol	79.6	118.8	8.0	49.2	77.6	1221
thymol	78.9	117.6	8.5	48.6	77.6	1073

BDE, bond dissociation enthalpy; IP, ionisation potential; PDE, proton dissociation enthalpy; PA, proton affinity; ETE, electron transfer enthalpy; EC_50_, half-maximal effective concentration.
